# Microfluidics for Antibiotic Susceptibility and Toxicity Testing

**DOI:** 10.3390/bioengineering3040025

**Published:** 2016-10-09

**Authors:** Jing Dai, Morgan Hamon, Sachin Jambovane

**Affiliations:** 1 Department of Electrical and Computer Engineering, Texas A&M University, College Station, TX 77843, USA; 2 Renal Regeneration Laboratory, VAGLAHS at Sepulveda, North Hills, CA 91343, USA; 3 David Geffen School of Medicine, University of California at Los Angeles, Los Angeles, CA 90095, USA; 4 Environmental Molecular Sciences Laboratory, Pacific Northwest National Laboratory (PNNL), Richland, WA 99354, USA; Sachin.Jambovane@pnnl.gov

**Keywords:** microfluidic platforms, antibiotic susceptibility, biofilm, bacterial persistence, non-growing but metabolically active (NGMA) bacteria, combinatorial effect, antibiotic toxicity, cell-on-a-chip, organ-on-a-chip

## Abstract

The recent emergence of antimicrobial resistance has become a major concern for worldwide policy makers as very few new antibiotics have been developed in the last twenty-five years. To prevent the death of millions of people worldwide, there is an urgent need for a cheap, fast and accurate set of tools and techniques that can help to discover and develop new antimicrobial drugs. In the past decade, microfluidic platforms have emerged as potential systems for conducting pharmacological studies. Recent studies have demonstrated that microfluidic platforms can perform rapid antibiotic susceptibility tests to evaluate antimicrobial drugs’ efficacy. In addition, the development of cell-on-a-chip and organ-on-a-chip platforms have enabled the early drug testing, providing more accurate insights into conventional cell cultures on the drug pharmacokinetics and toxicity, at the early and cheaper stage of drug development, i.e., prior to animal and human testing. In this review, we focus on the recent developments of microfluidic platforms for rapid antibiotics susceptibility testing, investigating bacterial persistence and non-growing but metabolically active (NGMA) bacteria, evaluating antibiotic effectiveness on biofilms and combinatorial effect of antibiotics, as well as microfluidic platforms that can be used for in vitro antibiotic toxicity testing.

## 1. Introduction

In recent years, emerging antimicrobial resistance (AMR) has become a global concern as antibiotics that are losing their effectiveness outpace new commercially available antimicrobial drugs, as most of the major pharmaceutical companies have stopped looking for new antibiotics, orienting their research and development (R&D) toward more profitable drugs [1]. As a consequence of the continued rise of resistance, it is expected that, by the mid-century, about 10 million people worldwide will die for AMR bacterial infection each year [2]. To overcome this prediction, policy makers have urged the development of new antibiotics and/or antimicrobial alternatives, or both [1]. Since, in a general manner, drug development is a long process, there is an urgent need for quick and accurate tools capable of evaluating the effectiveness and safety of antibiotics, thus, overcoming the paucity of new drugs in the R&D pipelines.

Recent progress in applied physics and chemistry has led to the development of microfluidic platforms that have the potential for conducting pharmacological studies [3]. By scaling down conventional macroscale systems to microscale, microfluidics appears as a solution for cheap, fast and accurate screening and testing of antibiotics and other drugs. The versatility of this technique allows the development of devices with task-specific architecture, such as simple microchannel-based systems for gradient formation [4], microchamber arrays for reduced cell population analysis [5], droplet-based system for high-throughput screening [6], and complex integrated systems such as microelectromechanical systems (MEMS) controlled real-time cell culture and monitoring [7]. The nature of laminar fluid flow within microscale enables the generation of spatial gradients of solutes, gases, and temperatures that mimic in vivo cell microenvironment [8].

In this review, we aim to summarize the recent developments of microfluidic platforms for antibiotic susceptibility and toxicity testing. We first report the progress of the microfluidic devices for evaluating antibiotic susceptibility, investigating bacterial persistence and non-growing bacteria with active metabolic activity, and assessing the effect of antibiotic combinatory therapy. Then, the application of microfluidic platforms that integrate cells and engineered tissues and organs for the determination of antibiotic side effects and toxicity are discussed.

## 2. Microfluidics for Antibiotic Susceptibility Testing

### 2.1. Bacterial Antibiotic Susceptibility Testing (AST) at the Single-Cell Level

**Figure 1:**
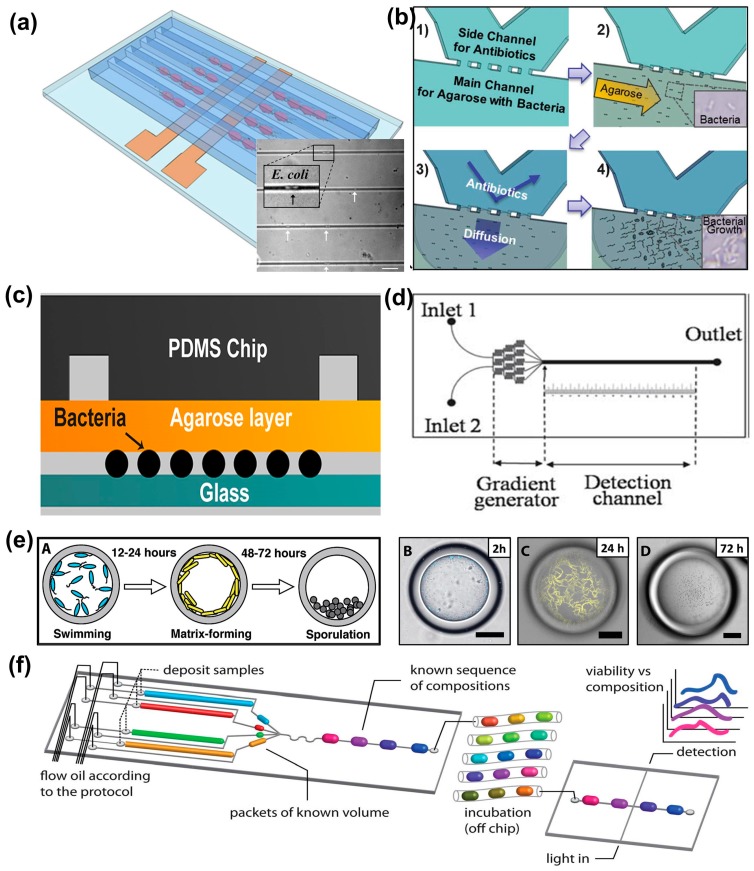
Microfluidic systems for assessing the effects of antibiotics on single cells, biofilms, and effects of multiple antibiotic combination. Single cell AST in (**a**) track channels (reprinted with permission from Reference [16]. Copyright 2013 American Chemistry Society); (**b**) agarose (adapted from Reference [17] with permission from Royal Society of Chemistry); (**c**) PDMS/membrane/coverslip sandwich structure (adapted from Reference [22]); Bacterial biofilm was treated with antibiotics in (**d**) microfluidic channels with a network of channels generating concentration gradient (adapted from Reference [50] with permission from Royal Society of Chemistry); (**e**) droplets where biofilm was formed at the interface of double and triple emulsion droplets (adapted from Reference [55] with permission from John Wiley and Sons); (**f**) Multiple antibiotic combination was generated in droplets by changing their compositions (adapted from Reference [61] with permission from Royal Society of Chemistry).

Antibiotic susceptibility testing (AST) is the most commonly used method to evaluate the effectiveness of single antibiotic or multiple antibiotic combinations to determine the most effective treatment for bacterial infections. Current AST techniques include disk diffusion, broth dilution, and commercially automated systems [9]. Although these techniques have been highly standardized, sufficient incubation time (16~20 h) is required for bacterial population to reach the minimum detectable growth level. Optical density (OD) measurement is commonly used to sense bacterial population with a limited detection of 10^7^ colony forming units (CFU/mL) [9,10]. By confining cells to a micrometer scale environment, microfluidics enables individual bacterial division at early stages, which ultimately reduces AST time. Recently, a number of studies have illustrated the potential of microfluidics for rapid AST, within a few hours, through the direct and indirect monitoring of cell growth, shown in Table 1. Direct optical imaging is simple and can be applied to all clinic isolates, while some indirect monitoring methods are limited due to the requirement of immunoassay, genetic modification, and sophisticated experimental setups. The short doubling time of bacterial cells enables the reduction of AST time. Therefore, the direct observation of single-cell division within the entire bacterial population is of great interest. Because of the high motility of many types of bacteria, immobilization of bacteria on-chip is necessary for time-lapse growth observation of a single bacterium. Single cell trapping has been shown to be feasible in droplets [11], microchambers [12], channels/tracks [13] or traps [14]. Additionally, Peitz et al. [15] and Lu et al. [16] demonstrated the capture and confinement of single *Escherichia coli* bacterium in microfluidic channels assisted by dielectrophoresis (DEP) (Figure 1a). Under such condition, *E. coli* AST was completed in 5 h and 1 h ([15,16], respectively) by monitoring the growth of single bacterium through time-lapse microscopy. In another example, Choi et al. [17,18] used agarose to encapsulate single bacterium cells on-chip. After gelation, culture media and antibiotics were supplied by perfusion through the gel (Figure 1b) and bacteria growth was monitored to determine the minimal inhibitory concentrations (MICs) of different types of antibiotics on *E. coli*, *Pseudomonas aeruginosa*, *Klebsiella pneumoniae*, *Staphylococcus aureus*, and *Enterococcus* spp. within only 4 h. However, tracking the growth of multilayer single bacterium cells in a 3D gel is challenging, thus, a polydimethylsiloxane (PDMS)-membrane-coverslip sandwich structure (Figure 1c) was developed to confine monolayer single bacterial cells [19,20,21]. Li et al. [22] integrated a gradient formation system to a similar chip assembly to determine the MIC and half maximal inhibitory concentration (IC_50_) of amoxicillin on *E. coli* in 4 h. PDMS microchannels that are perfused with drug solutions (in the “source” channel) and culture medium (in the “sink” channel) were used to establish a steady antibiotic concentration gradient in an agarose gel layer and antibiotic diffuses to the bacterial monolayer underneath. In contrast to determining antibiotic resistance in terms of bacterial growth, monitoring cell death that is facilitated by applying mechanical and enzymatic stress on a microfluidic chip can achieve rapid identification of antibiotic resistant strains within 60 min [23]. In addition to rapid AST, the MIC determined by microfluidic AST has been shown to be in good agreement with those by conventional methods for standard Clinical Laboratory Standard Institute (CLSI) strains [18,22,24,25]. Although there are a number of papers dealing with microfluidic rapid AST, few commercial systems have been developed for clinical use. The challenges of integration, standardization, economy of scale for mass production, and added value to the aimed applications impede the commercialization of microfluidic systems [26]. Currently, GRADIENTECH (Uppsala, Sweden) is developing a microfluidic-based rapid AST system, QuickMIC^TM^, which can generate phenotypic AST results in only 2 h [27].

### 2.2. Bacterial Persisters and Non-Growing but Metabolically Active (NGMA) Bacteria

A small fraction of bacterial population that tolerate antibiotics without genetic changes is called persistence bacteria (persisters). They play a major role in the recalcitrance of chronic and recurring infections to antibiotics [36]. The challenge of studying such cells by conventional bulky measurements highlights the importance of single-cell techniques such as single-cell trapping and time-lapse microscopy. Microfluidic devices with single-cell resolution capability have been employed to investigate the mechanism of persister formation and their growth dynamics to improve the understanding of bacterial survival strategies and advance the development of personalized antibiotic treatment of chronic diseases. A recent review [8] discussed the pioneer works that have been performed on a microfluidic chemostat [37], a microfluidic microchamber [38], and PDMS patterned grooves [39,40]. Recently, studies have been performed on the microfluidic chip with the PDMS-membrane-coverslip [20,41] or flow channel-membrane-coverslip sandwich structures [42,43]. The steady concentration gradient generated in the device from Gefen et al. [40] is not only used to determine the IC_50_ but also to evaluate the risk of spontaneous antibiotic tolerance to improve medication dosage. The detection and retrieval of persisters can be achieved in a femtoliter droplet array which is fixed on a hydrophilic-in-hydrophobic micropatterned surface [44]. The femtoliter droplets enable the trapping of single persister cells and make it easy to access and collect enclosed cells with a micropipette.

A subpopulation of cells that are non-growing but metabolically active (NGMA) may contribute to non-apparent infections that can recur after months or years of clinical latency. *Mycobacterium tuberculosis* is one well documented example [45]. The combination of microfluidics and sensing techniques has been utilized to identify these silent but potentially harmful bacterial reservoirs. For example, a PDMS-membrane-coverslip microfluidic device and a Förster resonance energy transfer (FRET)-based ATP biosensor have been used by Maglica et al. [46].

### 2.3. Bacterial Biofilm Antibiotic Susceptibility Testing

Bacteria in biofilms show much greater resistance to antibiotics than their planktonic counterparts, thus resulting in challenges to clinical treatments [47]. The features of microfluidics not only promote biofilm formation, but also benefit the assessment of influencing factors that contribute to biofilm growth and the effects of antibiotics on eradicating biofilms [48]. A Single channel microfluidic chip was utilized to perform biofilm antibiotic susceptibility test while antibiotic concentrations were prepared off-chip [49]. The on-chip antibiotic concentration gradient preparation was implemented by integrating a network of microfluidic channels (Figure 1d). For example, Kim et al. [50] and Matthew et al. [51] assessed the minimal biofilm eradication concentration (MBEC) against *P. aeruginosa* and *Staphylococcus pseudintermedius* and confirmed that the concentration required to eradicate bacterial biofilm is higher than that for planktonic bacteria. Sun et al. [52] developed nanoliter reactor arrays to form biofilm under static (no flow) condition and evaluated antibiotics against *E. coli* biofilm formation. Their results showed that tetracycline can facilitate the formation of long filamentous bacteria. In addition to optical imaging and fluorescence, electrochemical sensing [53] and electrochemical impedance spectroscopy (EIS) [54] have been used to monitor the activity of biofilm under antibiotic treatment to investigate biofilm antibiotic susceptibility. Recently, Chang et al. [55] demonstrated the formation of biofilm in microfluidic droplets, where biofilm was formed at the interface of double and triple emulsion droplets. The 3D culture of biofilm in droplets is more realistic over 2D culture in resembling biofilm growth in 3D extracellular environments. This approach allows the high-throughput screening of antibiotics for the eradication of biofilms (Figure 1e).

### 2.4. Combinatorial Effect of Antibiotics

The emergence of multidrug resistance among pathogenic bacteria urges the need for new strategies to combat antimicrobial resistance [56]. As one such strategy, combination therapy uses multiple drugs rather than individual drugs to treat bacterial infections. Mohan et al. [32] developed a multiplexed reactor array to investigate the combinatorial effect of two or four different antibiotics while significantly reducing AST time to 2~4 h. The network of microfluidic channels provides the capability of simultaneously generating a concentration gradient without tedious dilution steps, making it possible to assess the combinatorial effect of multiple antibiotics [57], anticancer drugs [58] and inhibitors [59]. Droplet-based microfluidic systems are capable of accommodating a large number of reactions in droplets and the composition of droplets can be programed by users with high reproducibility and homogeneity [60]. A series of T-junctions [61] or fluid manifolds [62] has been used to generate droplets containing multiple antibiotics in a concentration range, culture medium, and cells (Figure 1f). The droplets were generated in sequence and incubated after mergence to evaluate the combinatorial effect of multiple antibiotics against *E. coli* [61,63]. The classes of combinatorial responses such as additive, synergistic, or antagonistic effects were demonstrated for two or three antibiotic interactions by Churski et al. [61] as well as for binary and ternary mixtures of antibiotics and enzyme inhibitors by Cao et al. [63].

## 3. Microfluidics for in Vitro Drug Toxicity Testing

Like other drugs, antibiotics can be linked to various side effects such as nephrotoxicity [64] and hepatotoxicity [65]. Although their antimicrobial mechanisms of action are well understood, their effects on mammalian cells remain widely unclear and require extensive studies. In the last decade, microfluidic platforms have appeared as potential systems for pharmacokinetic and toxicity studies at the early stage of the drug development [3]. Two microfluidic systems have been developed: Cell-on-a-chip, which provides cytotoxicity information useful during the screening of drug candidates (dose response, mechanism of action); and organ-on-a-chip, which aims to give pharmacokinetics and toxicological information usually obtained at the later stage of drug development, from animal and human experiments.

### 3.1. Cell-on-a-Chip Technology

Cell-on-a-chip devices are usually composed of a cell culture area that can either be a simple channel [4,66] or a cell chamber [5,7], where cell monolayers are cultured and tested. Although, at this moment, we could not find any cytotoxicity studies of antibiotics on cell-on-a-chip devices, various reports have demonstrated the use of microfluidics with other drugs, including anti-cancer antibiotics [6,7].

**Figure 2:**
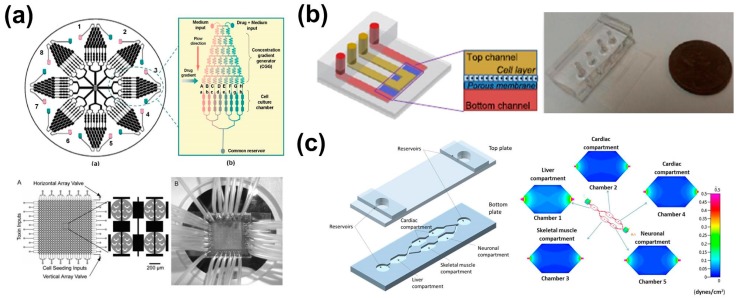
Microfluidic systems for toxicity studies on cells and tissues. (**a**) Cell-on-a-chip devices. Top: Schematic of an integrated microfluidic device for cell-based toxicity assay, consisting of eight uniform structure units (left). Each single structure unit (right close-up) containing an upstream concentration gradient generator and downstream parallel cell culture chambers (adapted from Reference [66] with permission from the Royal Society of Chemistry). Bottom: A microfluidic cytotoxicity array with 24 × 24 chambers (left). Each chamber (close-up) contains eight micro cell sieves for cell. Image of the chip with fluid interconnects (right) (adapted from Reference [5] with permission from Royal Society of Chemistry); (**b**) Schematic of the kidney-on-a-chip (left) and image of its actual size (right) (adapted from Reference [101] with permission from IOP Publishing); (**c**) Schematic of a body-on-a-chip for drug cytotoxicity testing (left) and shear-stress pattern on the 5 chambers of the system (right) (adapted from Reference [103]).

The versatility of microfluidics allows the development of various systems with different levels of complexity that fit the need of the experiment. For example, the coupling of a gradient maker with a microchannel, which could be considered as the simplest microfluidic cell culture system, enables fast cytotoxicity testing of drugs by exposing cells to a range of concentrations in a single experiment, whereas 24 well-plates were used to reproduce the experiment off-chip [4]. Additional systems arranged in parallel on one single chip can increase the throughput of the experiment by increasing the range of concentrations tested simultaneously. For example, Ye et al., integrated eight gradient makers with eight discrete cell culture chambers [66] (Figure 2a). The flexibility of the system allows for the testing of eight different concentration ranges of either the same molecule or eight different molecules simultaneously [66]. Other microfluidic functional units, such as individual microchambers, cell arresters and valves, can be integrated to protect cells from shear stress or to improve uniform cell distribution in the culture area, both being difficult to obtain within a simple microchannel. For example, to improve the homogeneity of cell distribution through 576 distinct circular microchambers, Wang et al., developed a microfluidic array that combines individual reaction chambers, cell arresters and multi-layer microfluidic valve systems [5]. The device, shown in Figure 2a, is composed of a 24 × 24 array of orthogonal microfluidic channels. At the intersection of two channels is a microchamber. Each microchamber contains eight U-shaped cell sieves for uniform cell distribution and can be compartmentalized by four pneumatic microvalves. Controlled actuation of the valves allows the introduction either of cells, by closing the channels in the “row” direction, or toxins by closing the channels in the “column” direction. Microfluidic systems can also be integrated into more complex systems that can monitor cells in situ in real-time [7]. For example, Caviglia et al., recently developed an integrated microfluidic cell culture and electrochemical analysis platform with a built-in fluid handling and detection system [7]. The device is composed of 4 microfluidic chambers integrated with 12 micro-electrodes, 4 peristaltic pumps, 4 reservoirs, and a potentiostat. The platform enables parallel electrochemical impedance spectroscopy measurement and optical detection for real-time monitoring and evaluation of cancer cell responses to anticancer drugs. The real-time monitoring of cell responses by the integration of an analytical tool into the device enables the study the time-dependent effects of different drugs, including the antibiotic anticancer drugs (doxorubicin and oxaliplatin) on cancer cells.

One critical step in the development of microfluidic systems for cell-based assays is to address the accessibility issues for the scientific and medical community members who are not familiar with microfluidic chips. One approach to this issue is to develop microfluidic devices with similar features to conventional cell culture systems, such as wells, which are compatible with standard automation apparatus while retaining the advantages of microfluidics. Toward the realization of such systems, Hamon et al., developed a microfluidic chip with an open culture area [67]. The chip is composed of 14 open micro-wells designed for conducting cell-based assays with the integration of a log-scale gradient generator. One main advantage of this system is that cells are directly introduced from the top of the open wells. It allows easier, faster, and a more homogeneous distribution of the cells across the microchambers than when introduced through a channel, without the use of cell traps and with fewer damaged cells. In another example, Lee et al., integrated their microfluidic chip into a conventional 96-well plate [68]. The microfluidic chip was bonded to the bottom of the plate such that laser-cut holes in the wells led directly into the microfluidic channels. The chip is composed of a cell culture unit separated from a culture medium channel by a 2-µm-tall barrier. As proof-of-concept, cytotoxicity of the anti-cancer drug etoposide was measured on HeLa cells cultured on the chip using commercial lactate dehydrogenase plate assays. The use of a 96-well plate interface makes the overall process very similar to cytotoxicity protocols performed by standard automation laboratories.

In all the previously described devices, the number of data points obtained from one single experiment is limited by the number of culture areas built into the device [69,70]. Droplet-based microfluidic devices can dramatically increase the number of data points (exceeding 10^6^) by performing high-throughput screening in a single experiment [71], with a volume as low as femtoliters, with high reproducibility and homogeneity [72,73,74]. The use of droplets as a reaction chamber presents various advantages. The physical and chemical isolation of the droplets eliminate the risk of cross-contamination. Moreover, droplets can be monitored [75], sorted [76,77,78] and digitally manipulated [79,80,81], stored and incubated off-chip [6,82,83], and transported and reintroduced into another microfluidic chip [6,84]. For example, Brouzes et al., developed a cytotoxicity screening method that uses three droplet-based microfluidics devices [6]. First, they generated a coded droplet library of drug compounds on an initial chip. In a second chip, each library droplet was merged with one cell-containing droplet. Merged droplets were then collected, incubated off-chip and re-injected into an assay chip to assess the specific effect of each compound on cells. IC_50_ value was obtained at a frequency 100 times faster than conventional high-throughput screening techniques.

### 3.2. Organ-on-a-Chip Technology

Cell-on-a-chip technologies overcome the limitations of the conventional cell culture systems by providing fast, cheap and easy-to-use tools for cytotoxicity screening. However, like conventional systems, they still use monolayers of target cells. Nevertheless, cells cultured in a monolayer have been shown to lose their phenotype [85] and may not be representative of their in vivo 3D counterparts [86]. Different strategies have been developed to create an on-chip 3D culture system that will improve cell phenotype, including by injection of a cell-loaded hydrogel into the microfluidic chip [87] and by the formation of cell spheroids on-chip when higher cell density models are required, e.g., tumor models [88]. For example, Ziolkowska et al., presented a chip integrated with micro-wells for the formation of HT-29 human carcinoma cell spheroids. Spheroids were cultured on-chip and the effects of the anticancer drug 5-fluorouacil on cell viability could be observed under a microscope.

The combination of tissue engineering, biomaterials, and microfabrication and microfluidics enabled the development of more accurate models that mimic human organs and tissues, termed organ-on-a-chip. Various organ-on-a-chip devices have been developed, including liver- [89], kidney- [90], lung- [91], intestine- [92], bone marrow- [93] and central-nervous-system-on-a-chip [94]. These devices are capable of replicating three important aspects of in vivo organs: 3D microarchitecture; functional tissue-tissue interfaces; and complex organ-specific mechanical and biochemical microenvironments [95,96,97,98]. Furthermore, they can be loaded with primary cells [99], or intact tissue [100]. When used with primary cells, they are capable of mimicking in vivo drug responses more closely than conventional culture conditions [99]. For example, Jang et al., described a human kidney proximal tubule-on-a-chip that can be used for nephrotoxicity assessment [90]. The chip is composed of a “luminar” flow upper channel separated from an “interstitial” lower compartment by a permeable membrane. Primary human proximal tubule cells were cultured on the upper channel. After the cells reached confluence, the channel was perfused with a culture medium at in vivo-like levels of shear stress. Under such conditions, cells recovered their normal, or close to normal, shape, polarization, and structure and enhanced kidney-specific functionalities compared to traditional Transwell cultures. In addition, cisplatin toxicity and Pgp efflux transporter activity measured on-chip were closer to the in vivo response than those measured in conventional systems. In another example, Kim et al. [101] introduced a kidney-on-a-chip (Figure 2b) to study the nephrotoxicity of gentamicin. The chip is composed of two superposed channels separated by a membrane. Madin–Darby canine kidney cells were loaded in the upper layer and cultured on the membrane under physiological shear-stress conditions. This system allows us to mimic the drug clearance for human bolus injection and compare it with conventional constant concentration and to show long low-level exposure cytotoxicity mechanisms. Intact tissues can also be used as an alternative to cells [100]. These systems present the advantage that cell–cell interactions, cell populations and biochemical components are present at physiological levels.

The next step toward the realization of a more systemic microfluidic system is to connect two or more (up to 14 [102]) organ-on-a-chip devices together to create a so-called “body-on-a-chip” or “human-on-a-chip”. These systems aim to reproduce in vivo pharmacokinetic, pharmacodynamic and ADME (absorption, distribution, metabolism, and excretion) profiles, or to investigate toxicity involving multiple organs or inter-organ interactions. Although no toxicity studies have been reported for antibiotics, using the body-on-a-chip technology, numerous studies showing the possibility of using this technique for toxicity testing of other drugs have been reported. For example, Oleaga et al., introduced a four-organ system that mimics the human response to five different drugs for 14 days [103]. The system, shown in Figure 2c, is composed of a gravity driven flow system that perfuses culture medium through five interconnected chambers (two cardiac compartments, one liver compartment, one skeletal muscle compartment and one neuronal compartment). The electrical and mechanical responses of the system to the five drugs were in agreement with published toxicity results from both human and animal data.

## 4. Conclusions and Outlook

The advancement of microfluidics facilitates the assessment of antibiotic effectiveness on treating infections as well as the toxicity and side effects of drugs on human tissues. The capability of manipulating fluids and the flexibility on geometries and materials makes microfluidic devices a versatile tool for almost every step of drug discovery and development. Proof-of-concepts demonstrated the possibility of making microfluidic devices accessible to the scientific community, but efforts still need to be made to render microfluidic platforms fully compatible with existing sample preparations and imaging apparatuses. We expect that microfluidic devices will serve as an excellent tool to overcome the paucity of new antimicrobial agents in the R&D pipelines and facilitate the clinical treatment of bacterial infections.

## Figures and Tables

**Table 1:** Direct and indirect cell growth monitoring for microfluidics-based rapid antibiotic susceptibility testing (AST).

Growth Monitoring Methods		Description	AST Time [Reference]	Advantage/Limitation
Direct	Optical imaging	Number of cells	2 h [28], 4 h [29], 5 h [15], 1 h [16], 3 h [25]	All clinic isolates
		Area of cells	4 h [17], 4 h [22]	
		Greyscale imaging of cells	2.5~4 h [24]	
Indirect	Fluorescence	Fluorescent signal of bacteria-microbead complex	4~8 h [30]	Immunoassay is required
		GFP expression of bacterial strains	7.5 h [31], 2~4 h [32]	Only molecularly engineered strains
	Bioluminescence	ATP bioluminescence of bacteria-antibodies complex	3~6 h [33]	Immunoassay is required
	Magnetics	Magnetic beads rotation rate which is inversely proportional to bacterial mass	30 min [34]	Immunoassay & external rotational magnetic field are required
	pH	pH changes due to the accumulation of metabolic products	2 h [35]	All clinic isolates
